# Rho GTPases and Regulation of Cell Migration and Polarization in Human Corneal Epithelial Cells

**DOI:** 10.1371/journal.pone.0077107

**Published:** 2013-10-10

**Authors:** Aihua Hou, Li Xian Toh, Kah Hui Gan, Khee Jin Ryan Lee, Edward Manser, Louis Tong

**Affiliations:** 1 Ocular Surface Research Group, Singapore Eye Research Institute, Singapore, Singapore, Singapore; 2 RGS Group, Institute of Medical Biology, A, Star, Singapore, Singapore; 3 Singapore National Eye Center, Singapore, Singapore, Singapore; 4 Duke-NUS Graduate Medical School, Singapore, Singapore, Singapore; 5 Yong Loo Lin School of Medicine, National University of Singapore, Singapore, Singapore, Singapore; Karolinska Institutet, Sweden

## Abstract

**Purpose:**

Epithelial cell migration is required for regeneration of tissues and can be defective in a number of ocular surface diseases. This study aimed to determine the expression pattern of Rho family small G-proteins in human corneal epithelial cells to test their requirement in directional cell migration.

**Methods:**

Rho family small G-protein expression was assessed by reverse transcription-polymerase chain reaction. Dominant-inhibitory constructs encoding Rho proteins or Rho protein targeting small interfering RNA were transfected into human corneal epithelial large T antigen cells, and wound closure rate were evaluated by scratch wounding assay, and a complementary non-traumatic cell migration assay. Immunofluorescence staining was performed to study cell polarization and to assess Cdc42 downstream effector.

**Results:**

Cdc42, Chp, Rac1, RhoA, TC10 and TCL were expressed in human corneal epithelial cells. Among them, Cdc42 and TCL were found to significantly affect cell migration in monolayer scratch assays. These results were confirmed through the use of validated siRNAs directed to Cdc42 and TCL. Scramble siRNA transfected cells had high percentage of polarized cells than Cdc42 or TCL siRNA transfected cells at the wound edge. We showed that the Cdc42-specific effector p21-activated kinase 4 localized predominantly to cell-cell junctions in cell monolayers, but failed to translocate to the leading edge in Cdc42 siRNA transfected cells after monolayer wounding.

**Conclusion:**

Rho proteins expressed in cultured human corneal epithelial cells, and Cdc42, TCL facilitate two-dimensional cell migration *in-vitro*. Although silencing of Cdc42 and TCL did not noticeably affect the appearance of cell adhesions at the leading edge, the slower migration of these cells indicates both GTP-binding proteins play important roles in promoting cell movement of human corneal epithelial cells.

## Introduction

The cornea epithelium ensures appropriate light transmission and refraction for clear vision and also protects the eye against environmental insults, physical trauma, and chemical injury [1-3]. Cornea becomes hydrated and opacified when the epithelial barrier is lost; initiation of wound healing requires migration of epithelial cells over the corneal stroma to restore the continuity of corneal epithelium. Corneal epithelial cells express a variety of proteins not found in other epithelial cells such as keratin 12 [4]. The differentiation of these cells is also related to the migration process, since ‘progenitor’ cells migrate centripetally from the limbus towards the center. For these reasons, the molecular control of corneal epithelial cell migration deserves further study, especially for processes that are known to be cell type specific.

The Rho family small G-proteins are involved in most aspects of cell motility through ability to promote reorganization of the cytoskeleton [5,6]. Rho-family proteins are also needed for cell adhesion complex assembly which requires actomysoin contractility [5,6]. Rac proteins are widely used to facilitate cell expansion on two-dimensional (2D) matrices through the formation of lamellipodia ruffles; human corneal epithelial cells are reported to require Rac1 for optimal migration [7]. The protein Cdc42 is needed to direct cell polarization in many cell types [5,8] and acts through its kinase effector myotonic-dystrophy-kinase-related CDC42-binding kinase (MRCK) to control the actomyosin filaments in the lamellipodia [9,10]. Alterations of Cdc42 expression in rabbit corneal epithelial cells could impact wound repair [11]. RhoA regulates corneal epithelial cell migration and focal adhesion formation through a related kinase family Rho kinase (ROCK) is the mostly well-studied in corneal epithelial cells [12-17]. Inhibiting ROCK with the compound Y-27632 promotes basal and heparin-binding EGF-like growth factor (HB-EGF)-enhanced human corneal epithelial cell migration and adhesion to matrices [16]. Furthermore, it was found that Y-27632 disrupts E-cadherin- and beta-catenin-mediated cell-cell junctions [18]. Rho proteins can be activated by Rho activators (guanine nucleotide exchange factor, GEF) [5]. Depletion of the Rac1 and Cdc42 GEF β-Pak-interacting exchange factor (β-Pix) in human cornea epithelial cells has been reported to inhibit their migration on fibronectin in an in vitro model of wound healing [19]. However, the GEF for RhoA responsible for stabilizing cornea epithelial junctions has not been found. The role of potential downstream G-proteins Cdc42 and TCL has not been studied in this context.

During cell migration, multiple events are coordinately regulated, including: polarization to the direction of movement, reorganization of cytoskeleton and remodeling of cell adhesion [20]. Cells are polarized in response to environmental and molecular cues like chemotaxis [21,22]. After polarization is established, the microtubule organizing center and Golgi body re-align toward the wound or direction of sheet migration [23,24]. Polarization of cells during cell movement has been intensively evaluated in standard fibroblasts and epithelial cell lines but this phenomenon can be quite cell-type specific [23,25,26]. Cdc42 has been shown to play a central role in cell polarity in a wide range of biological contexts [8]. 

RhoA and Rac1 have been shown to play important roles in corneal epithelial cell migration and proliferation [7,11-19]. Recently, Cdc42 also has been reported as an important regulator during corneal epithelial wound repair [11]. These studies on corneal epithelial migration have not extended to other Rho family members. In this paper we survey the expression of Rho GTPase members in human corneal epithelial cells, and assess the potential role of each member in cell movement in an *in vitro* context. Using dominant inhibitory and siRNA approaches, we found that small G-proteins TCL and Cdc42 are significantly expressed in this cell type and are needed for optimal cell migration.

## Materials and Methods

### Cell Culture

Human corneal epithelial Large T antigen (HCET) cells used in this study are non viral shedding SV40-immortalized human corneal epithelial cells [27]. HCET cells were cultured in Dulbecco’s minimum essential medium (DMEM)/F12 supplemented with 5% fetal bovine serum (FBS) at 37°C in 5% CO_2_ incubator. Cells were sub-cultured at 80% confluence by being trypsinized in 0.05% trypsin. Fresh human corneal tissue were obtained from Singapore Eye Bank (http://app.sgdi.gov.sg/listing.asp?agency_subtype=dept&agency_id=0000011126). Primary limbal/corneal epithelial cells were cultured from cadaveric human limbal explants as previously described [28].

### Reverse Transcription-Polymerase Chain Reaction (RT-PCR)

Reverse transcription-PCR was performed as previously published [29]. In brief, RNA isolated from HCET, HeLa and primary limbal cells was reverse transcribed using Invitrogen Superscript III kit. The cDNA was amplified using the respective primers (Table S1 in File S1).The amplified products were run on 2% agarose gel and stained with ethidium bromide and imaging was performed as described previously [29].

### Transfection by Electroporation

Electroporation was performed using the Invitrogen Neon™ Electroporation transfection kit according to a previous protocol used [30]. Briefly, HCET cells (1x10^6^) were suspended in 120μl of solution R before adding 1μg of dominant-negative plasmid DNA or 40pmol of siRNA. The HCET cells-solution R- DNA or siRNA mixture was then electroporated in 4ml of solution E2 at 1300V, 30ms in a single pulse. After that, the electroporated HCET cells were mixed with 1ml of media, and seeded into wells of 12-well plates. One hundred microliters of cells were taken and seeded into 1 well of the 2-well culture inserts (Ibidi GmbH, Martinsried, Germany) which were placed in a 12-well plate. Rho dominant negative plasmids, designed to inhibit upstream Rho activators, were constructed by Dr. Edward Manser and Rho siRNA were purchased from Dharmacon Inc. (Chicago, IL). Details of the siRNA used in this study were in Table S2 in File S1. Allstar negative control siRNA (Qiagen) was used as control. Transfection efficiency of dominant-negative plasmids was evaluated by observing green fluorescent protein (GFP) signal under fluorescence microscope. siRNA inhibition efficiency was detected by western blot 48 hrs after transfection as described previously [31], and the intensity of western blot bands were measured by ImageJ version 1.45 (National Institute of Health, USA). 

### Cell Migration Assay

Dominant negative or siRNA transfected cells (1x10^6^) were cultured in DMEM/F12 with 5% FBS for 24hrs and then subjected for cell migration assay. For *in vitro* scratch wounding assays, the monolayer of HCET cells in 12-well plates was physically wounded with a 1000µl pipette tip. As for no-traumatic assay, culture inserts (Ibidi GmbH, Martinsried, Germany) were used as barriers to create linear/rectangular gaps (500μm+ 50μm) in sheets of HCET cells without physical wounding. The insert was then removed using a pair of sterilized forceps with one swift pull. In both experiment conditions, the HCET cells were washed with phosphate buffered saline (PBS) once and cultured in DMEM/F12 with 5% FBS. The area devoid of cells was imaged at 2-hourly intervals until the cells from both sides of the bare area merged. The Nikon Eclipse TS100 bright field microscope (Nikon, Singapore) equipped with Nikon DS-Fi1 camera was used to visualize and image cultured cells. The NIS-Elements F.300 software was used to acquire the imaging data.

### Quantification of Wound Size

The software ImageJ version 1.45 (National Institute of Health, USA) was used to document the width of the remaining gap every 2 hours. Each wound was measured six times and the average was taken. Independent duplicates were measured similarly. 

### p21-activated kinase 4 (PAK4) immunofluorescence Staining

siRNA transfected cell were incubated overnight, and then starved in DMEM/F12 with 1% FBS for 24hrs. Monolayer of cells was wounded with a pipette tip. Cells were incubated in fresh DMEM/F12 with 5% FBS for 1hr and immunofluorescence was performed as previously described [31]. Briefly, Cells were washed with PBS for three times and fixed with 100% cold methanol for 10 minutes in -20°C. Fixed cells were blocked with 10% goat’s serum for 10 minutes at room temperature, and incubated with Anti-PAK4 (Cell Signaling, cs3242) as described previously [32] diluted in 0.5% PBS/Triton X-100 and incubated overnight at 4°C. After that, cells were washed with 0.1% Triton X-100 for three times, 5 minutes each, and then incubated with secondary antibody and 4',6-diamidino-2-phenylindole (DAPI) diluted in 0.5% Triton X-100 for 45 minutes at room temperature. The immunostained cells were then mounted on Fluorsave™ reagent (Merk Millipore, MA, USA) and visualized with the Olympus FV1000 upright confocal microscope and acquired using the FV10-ASW 3.0 Viewer software. 

### Polarization assay

HCET cells were transfected with control siRNA, siRNA targeting Cdc42 or siRNA targeting TCL individually by electroporation method described above. For polarization assay in scratch wounding conditions, the transfected cells (0.8 x 10^6^) were seeded on 8-well chamber slides and incubated overnight. For polarization assay in non-traumatic cell migration conditions, transfected cells (1 x 10^5^) were seeded into culture insert which was firmed stuck on a 22 × 22 mm glass coverslips, the coverslip was in a well of 6-well plate and incubated overnight. In both conditions, cells were then starved in DMEM/F12 with 1% FBS for 24 hours. For cells cultured in 8-well chamber slides, a single scratch was made using a 10µl pipette tip across the confluent monolayer. For non-traumatic conditions, culture insert was removed. After that, cells were incubated in fresh DMEM/F12 with 5% FBS for one hour, followed by immunofluorescence staining for Golgi body using anti-Golgi Matrix protein GM130 (Epitomics, Inc & EP892Y) as described in ‘PAK4 Immunofluorescence Staining’. 

### Polarity scoring

This method of documenting cell polarization was previously published [33,34]. Briefly, imaginary lines were drawn from center of nucleus outwards dividing the cell into 3 equal sectors of 120 degrees. If the stained Golgi body were located primarily in the cytosolic sector facing the wound or direction of migration, the cell was considered as polarized. At least 100 cells near wound edge were randomly selected and assessed and the percentage of polarized cells determined as described under ‘Statistical analyses’.

### Statistical Analyses

Statistical analysis on the average wound sizes measured with ImageJ was done using General Linear Model (GLM) on SPSS for Windows version 17-20. Within each well 6 measurements were averaged, and 2 independent replicates were performed per condition. In GLM analysis, the dependent variable was the remaining width of gap at 2-hour time points in microns. The between group variable was the type of siRNA transfected into the cells. Post Hoc tests with adjusted p values were performed to assess for statistically significant difference between each siRNA against control. 

## Results

**Figure 1:**
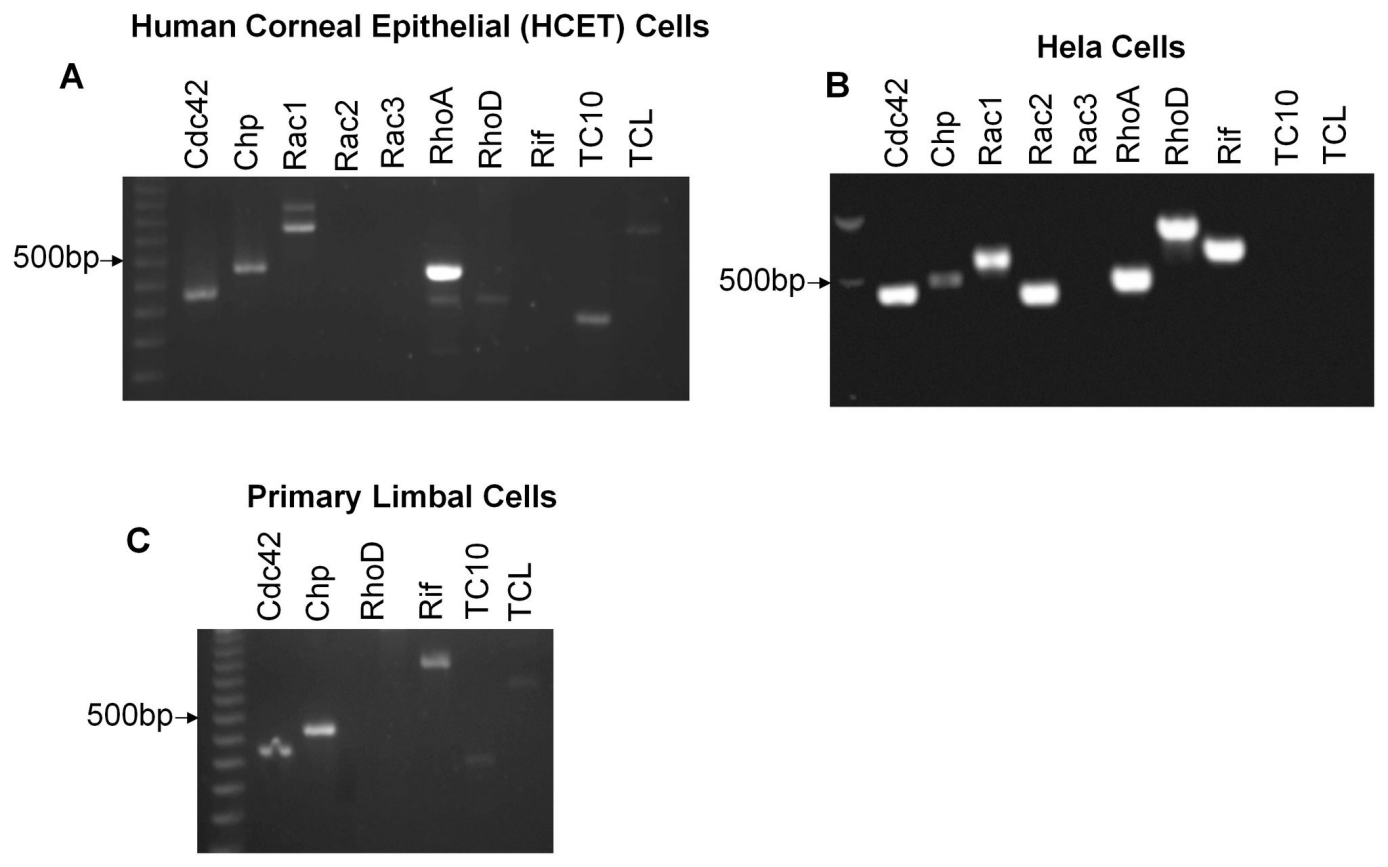
Reverse transcription-PCR results showing the detection of various members of Rho proteins in human corneal epithelial cells (A), HeLa cells (B) and primary limbal cells (C).

Rho proteins are critical drivers of cell migration in culture and *in vivo* and act by engaging a wide variety of effector proteins that can lead to changes in the actin cytoskeleton [5,35]. The small GTP binding proteins RhoA, Rac1 and Cdc42, are conserved across metazoans and also found in many protozoa [36], however vertebrates contain a number of less studied Rho-family G-proteins such as Chp, Wrch, Rif, RhoD, TCL and TC10. Chp and Wrch are Cdc42-like GTPases that lie downstream of Wnt signaling in development [37]. We used RT-PCR to exam the expression of human Rho proteins in immortalized HCET cells (Figure **1A**) using HeLa cells as a positive control for a number of the primer pairs (Figure **1B**). These cells did not appear to express Rac2, Rac3, RhoD or Rif, but expressed Chp, TC10 and TCL which are not found in invertebrates. The expression of Chp, TC10 and TCL mRNAs was confirmed in primary limbal epithelial (Figure **1C**); these experiments indicated Rif is also expressed which was not found in the HCET cell line. In summary, HCET cells express a variety of Rho proteins, where RhoA mRNA levels appeared dominant.

### Inhibition of Cdc42 and TCL impedes wound closure rate

**Figure 2:**
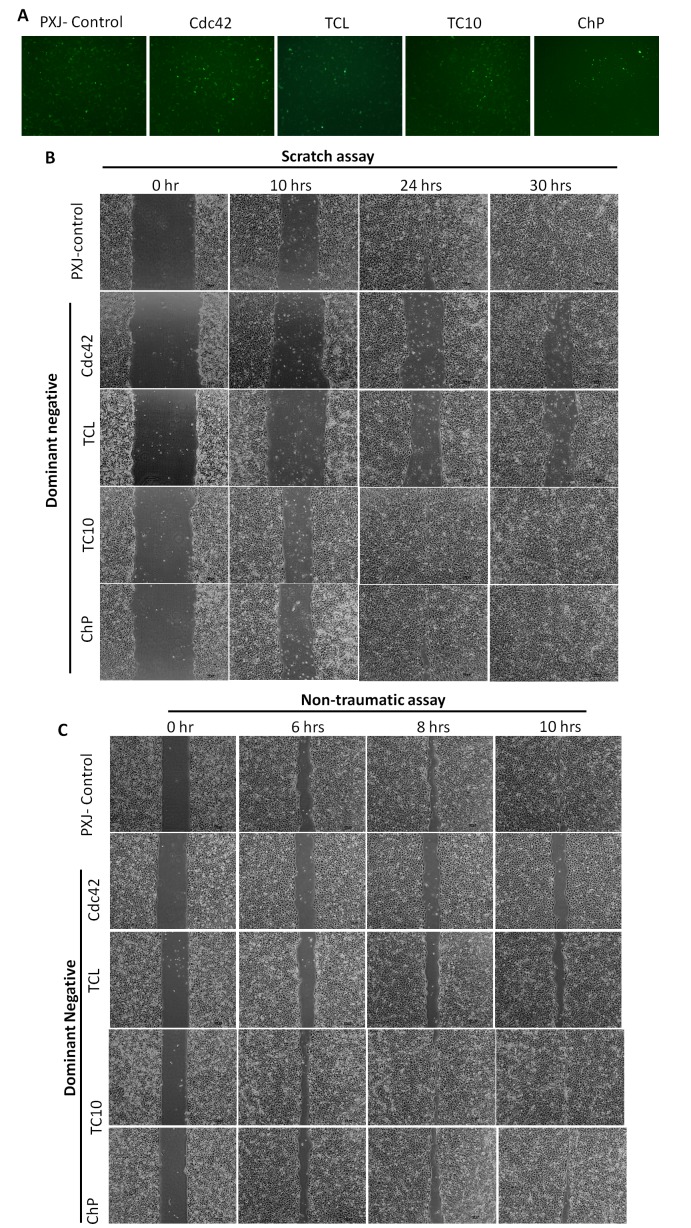
Inhibition of Cdc42 or TCL by dominant-negative plasmids delayed HCET cell migration. A. Transfection efficiency of control plasmids and dominant negative plasmids were similar when evaluated by observing GFP under fluorescence microscope. B. *In* *vitro* scratch wounding assay performed on human corneal epithelial cells transfected with Cdc42, TCL, TC10 or Chp dominant-negative plasmids. Control plasmid transfected cells and cells transfected with TC10, Chp dominant-negative plasmids closed wound faster than Cdc42 or TCL dominant-negative plasmids transfected cells. C. Non-traumatic cell migration assay performed on human corneal epithelial cells transfected with Cdc42, TCL, TC10 or Chp dominant-negative plasmids. Results were similar with scratch wounding assay.

To study the potential role of the Rho GTPases that are expressed in HCET in cell migration, we expressed dominant-negative versions of Rho GTPases in these cells using electroporation to achieve high transfection efficiency (we tested Cdc42, TCL, Chp, RhoD, Rac1 and TC10). These proteins were tagged with GFP, which allowed us to confirm similar transfection efficiency for the various GTPases, and overall level of expression was similar for all the plasmids used (Figure **2A**). Twenty-four hours after transfection, the cells were tested for migration in a standard ‘monolayer scratch’ assay, most constructs failed to affect cell migration rate under these conditions as shown for dominant inhibitory Chp or TC10 (Figure **2B**). Notably, closure was delayed in cells transfected with dominant-inhibitory version of Cdc42 or TCL plasmids (Figure **2B**). A non-traumatic migration assay yielded similar results suggesting that cell migration is affected whether or not cells are stressed (Figure **2C**). Thus Cdc42 and TCL, but not Rac1, appear to be important in driving coordinated cell migration of HCET monolayers. 

**Figure 3:**
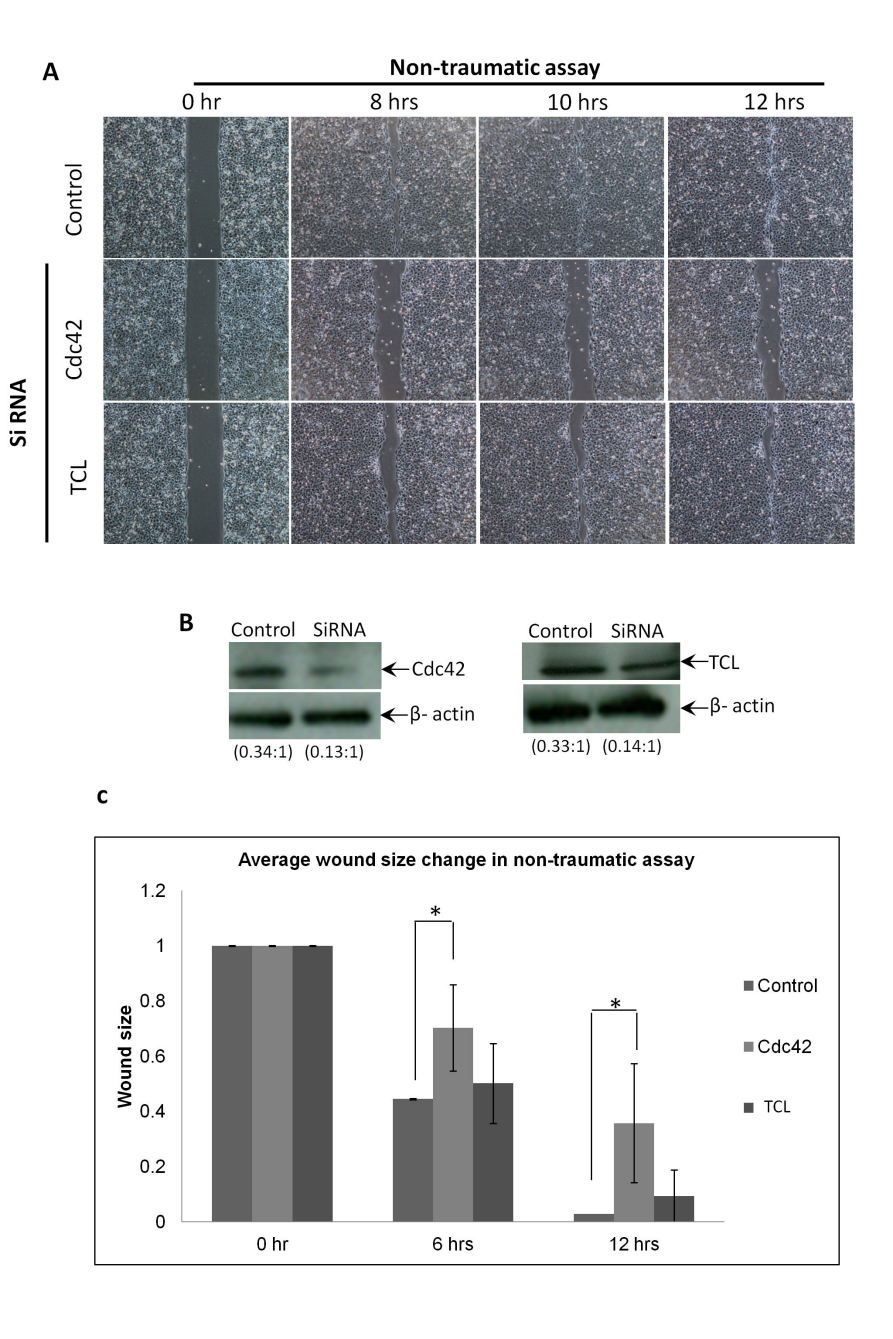
Inhibition of Cdc42 or TCL by siRNA delayed Non-traumatic cell migration. A. Non-traumatic cell migration assays performed on human corneal epithelial cells transfected with siRNA. Migration of cells transfected with siRNA targeting Cdc42 or TCL was delayed compared to control siRNA transfected cells. B. Western blot showed that Cdc42 or TCL were inhibited efficiently by individual siRNA 48hrs after transfection. The ratio of Cdc42 and TCL to loading control were shown. C. Chart of the averaged wound size change against time. Results shown are for siRNA transfected non-traumatic cell migration assay over 12 hours. Wound closure rate in cells transfected with siRNA targeting Cdc42 were significantly slower than control cells. *p<0.05.

To confirm these results using a complementary method, we subjected the cells to RNA interference and used the non-traumatic migration assay. Again both Cdc42 and TCL knockdown reduced cell migration (Figure **3A**). Western blot confirmed that Cdc42 and TCL targeting siRNA significantly reduce the protein level of Cdc42 and TCL (Figure **3B**). Figure **3C** depicts the region of the ‘wound’ remaining in such an assay: the delay of ‘wound’ closure (or cell migration) was more pronounced using siRNA against Cdc42 than TCL (Table S3 in File S1), but this might reflect that the TCL siRNA is less effective. That being the case we decided to focus on the potential players downstream of Cdc42 that might affect the migration of HCET cells.

### Silencing of Cdc42 and TCL affect cell polarization

**Figure 4:**
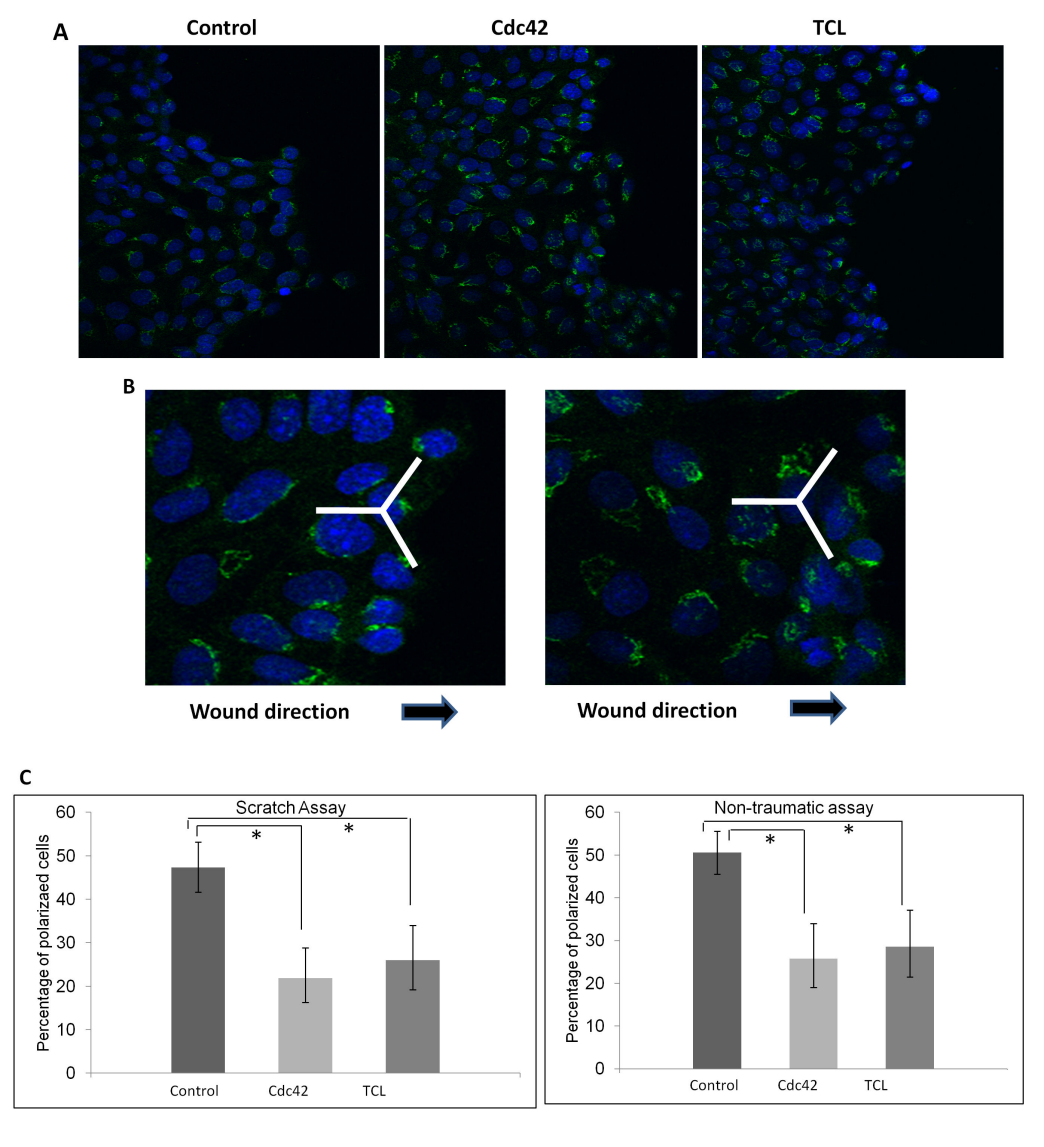
Cdc42 and TCL are required for HCET cell polarization. A. SiRNA transfected HCET cells stained with Golgi body (GM130) (green). Nuclei were counterstained with DAPI (blue). B. Typical cells that were polarized (left) and not polarized (right) were shown. C. Percentage of polarized cells. Percentage of polarized cells among cells transfected with Cdc42 or TCL siRNA were significantly lower than control siRNA transfected cells in both scratch assay and non-traumatic assay. * P<0.05.

Figure 4A and 4B showed the representative images after Golgi body staining in siRNA transfected cells and control cells. Figure **4C** showed the proportion of polarized cells in both scratch assay and non-traumatic conditions. In both conditions, the proportion of polarized cells in control cell was significantly higher compared to cells transfected with siRNA targeting Cdc42 or TCL respectively.

### Silencing of Cdc42 alters PAK4 localization

**Figure 5:**
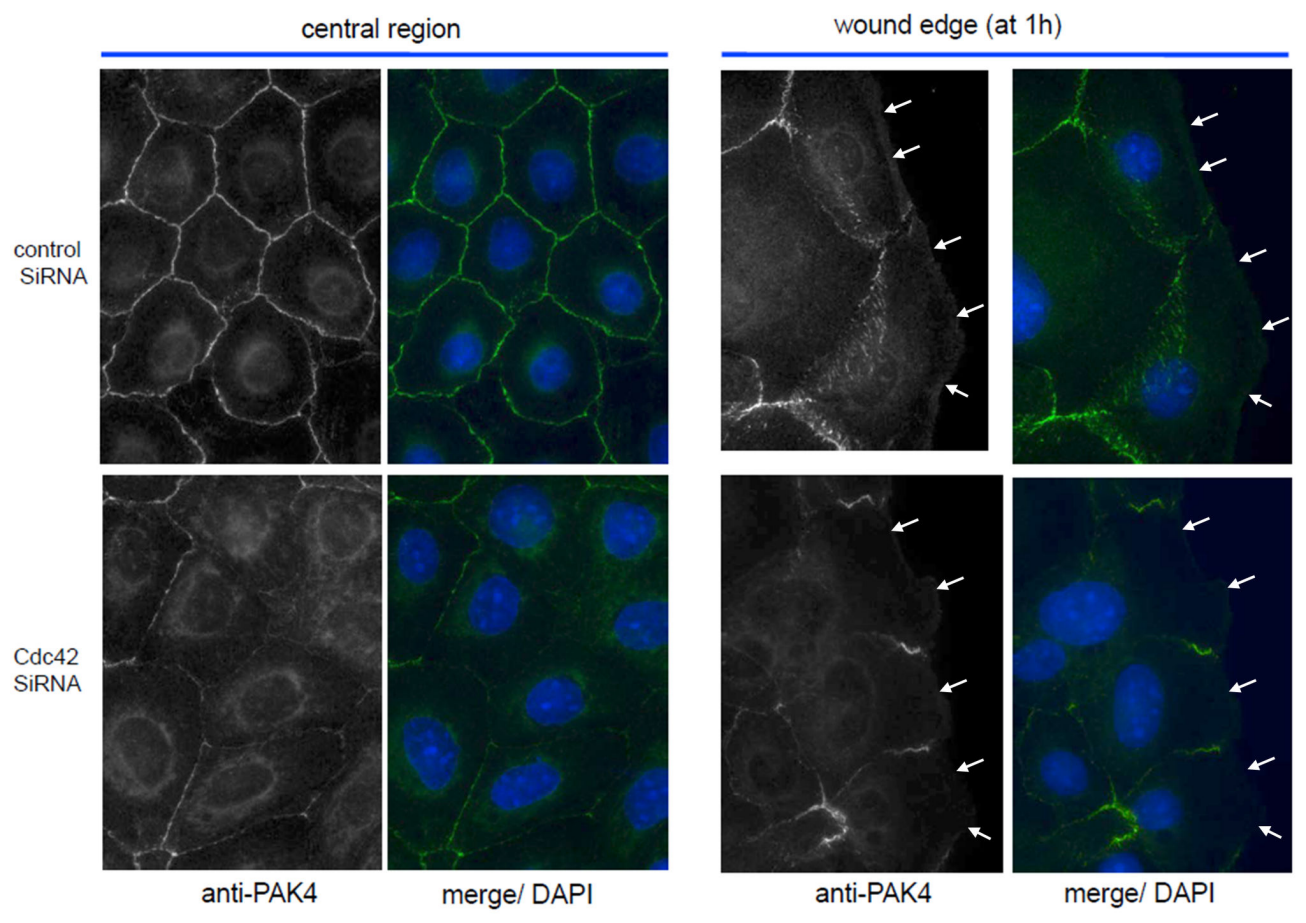
Inhibition of Cdc42 by siRNA impaired PAK4 localization. HCET cells transfected with control siRNA or Cdc42 siRNA were wounded and allowed to migrate for 1hr. Immunostaining for PAK4 were performed. Green: PAK4; Blue: Nulcei. PAK4 junction localization was destroyed at both central region and wound edge. Arrows point to leading edge.

In order to understand how Cdc42 might be operating, we investigated the subcelllular distribution of MRCK, neuronal Wiskott–Aldrich Syndrome protein (N-WASP) and PAK4 which are Cdc42-specific effectors involved with actin organization [38-40]. Only PAK4 was found to be perturbed by Cdc42 siRNA whereas anti-N-WASP immuno-staining was predominantly in the peri-Golgi region with and without Cdc42 siRNA. MRCK remained associated with the lamella region in migrating cells but likewise unperturbed (data not shown). The anti-PAK4 immuno-localization indicated PAK4 is found predominantly associated with cell-cell junctions, however in Cdc42 siRNA transfected cells this localization was greatly reduced (Figure **5**). In addition to loss of junction PAK4, we noted that the PAK4 was not found to be as strongly localized at the leading edge of migrating cells when treated with Cdc42 siRNA (Figure **5**). Given that PAK4 has been shown to play a key role in migration in a number of cell types [41-44], we conclude that in HCET cells the loss of Cdc42 leads to a failure to properly localize and activate PAK4. We have recently shown that PAK4 activity is upregulated about 5-fold by Cdc42 *in vivo* [32].

## Discussion

This study screened expression and function of Rho family members in corneal epithelial cell migration, showed that Cdc42 and TCL play important roles in regulating the rate of cell migration in a typical ‘wound’ closure assay. Since cell cycle progression and cell proliferation (Figure **S1** and Figure **S2**) were unaffected by inhibiting these Rho proteins, the inhibition of wound closure is unlikely due to effects on cell division/ proliferation. The low number of proliferating cells in these assays is consistent with significant contact inhibition in confluent HCET monolayer as part of normal growth control [45]. Further, cell polarization was affected by decreased Cdc42 and TCL. To the best of our knowledge this is the first report showing that TCL expression alteration regulates corneal epithelial cell migration and polarization.

Cdc42 has been reported to affect cell migration rates as well as cell polarization in response to chemotactic cues [5]. A study in rabbit corneal endothelial cells has demonstrated that over-expression of Cdc42 facilitated migration, although in their hands dominant negative Cdc42 transfection did not affect cell migration [46]. Another study in mouse embryonic fibroblasts demonstrated that Cre-mediated excision of Cdc42 resulted in reduced fibroblast migration which was fully restored with Cdc42 re-expression [47]. During the submission of this paper, Pothula et al reported the importance of Cdc42 in corneal epithelial wound healing [11]. The relative importance of different Rho GTPases could change during specific developmental stage [48], a prominent role for Cdc42 in cell migration has only been observed in a few cell lines [46,47],[49][50], and sets the stage for further studies of how Cdc42 and TCL each contribute to this process.

The role of TCL in cell migration is largely unexplored although siRNA directed against TCL was reported to decrease umbilical vein endothelial cell migration [51]. TCL is prominently expressed in endothelial cells [51,52] and studies of TCL knockout mice indicate it is important for neonatal retinal vascularization [53]. In fibroblasts which express Cdc42, Rac1, Rac3, TC10, TCL and Wrch, apparently only loss of Cdc42 prevents efficient chemotaxis of cells toward platelet-derived growth factor-BB (PDGF-BB) [54]. In these fibroblasts, Cdc42 knockdown cells exhibited ~25% reduction in the speed of migration compared to controls, while Rac1 knockdown had no effect. 

Our data showed that Rac1 inactivation had no obvious effect on the motility of human cornea epithelial cells, even though we are confident that the dominant inhibitory Rac1 protein was expressed at levels similar to Cdc42N17. Our result is hence apparently different from results reported by Kimura et al [7]. Kimura and colleagues’ work indicated that Rac1 is necessary for the promotion of HCE cell motility by fibronectin. In their report, Motility of HCE cells transfected with Asn17Rac1 vector was reduced greatly compared with cells transfected with a control vector. This apparent discrepancy may be related to the use of different extra-cellular matrices. Kimura et al pre-coated plates with fibronectin, our experiment carried out using uncoated culture vessel. Also, Kimura et al observed migration of randomly moved cells, while the current study observed directional cell migration.

Cdc42 is more commonly required for the polarization of cells, across many studies with different cell lines and assays [8,23,24]. Loss of Golgi reorientation is a common effect that can be observed in cell culture [24,26]. In this pathway, the downstream effector proteins needed for centrosome/Golgi re-orientation in monolayer scratch assays include PAR6 [55] and MRCK [56]. We found that knocking down Cdc42 or TCL had no obvious effect on the organization of focal adhesion at wound edge nor did PAK4 localize to these structures (data not shown). In umbilical vein endothelial cells loss of TCL is reported to increase focal adhesion number and stress fibers [57]. Activation of Cdc42 can cause focal adhesion redistribution in HeLa cells [58] in fibroblasts [59] and human pulmonary endothelial cells [60]. Interestingly, silencing of RhoA and -C isoforms in corneal epithelial cells resulted in a reduction in paxillin levels [17]. PAK4 is Cdc42 specific [61] and it is commonly believed over-expression of PAK4 can increase MDCK cell migration [62]. Cdc42 may therefore facilitate HCET cell migration via a PAK4 pathway. Cdc42 and TCL are presumably playing non-redundant roles. These pathways could involve effects on local actin assembly, or actin-associated molecular motors [6]. In addition, it is suggested Cdc42 acts through the Par 6-aPKC complex to regulate microtubules [23,24]. 

Epithelial cells typically undergo collective migration over the injured or open area [63,64] to restore barrier function. In the cornea, the speed of wound healing is functionally important because it can limit vision recovery. Currently, a commercial pharmacological drug (Y-27632) targeting ROCK, an effector of RhoA is used for the treatment of corneal endothelium disorders [65,66]. Given the antagonism between Cdc42 and RhoA [46,67], it is possible pathways that activate Cdc42 or TCL proteins function to antagonize RhoA and increase migration. 

In summary, Cdc42 and TCL have a facilitatory role in 2-D cell migration *in-vitro* in cultured human corneal epithelial cells. Silencing of Cdc42 or TCL proteins did not noticeably affect localization of cell adhesion but it seems likely these two molecular switches act on different pathways to facilitate cell movement where Cdc42 acts upstream of PAK4.

## Supporting Information

File S1 **File includes Supplementary Material and Method and Tables S1, S2, and S3.**
(DOCX)

Figure S1 **Cell cycle Assay.** A. HCET cells transfected with control plasmid, Cdc42 dominant-negative plasmid or TCL dominant-negative plasmid were stained with Guava Cell Cycle reagent and analyzed by Guava flow cytometry system. Percentages of cells at different cell cycle phase were shown. B. HCET cells transfected with control siRNA, Cdc42 targeting siRNA or TCL targeting siRNA were subjected to cell cycle assay as in A. In both conditions, cell cycles were not affected by Cdc42 or TCL inhibition.
(TIF)

Figure S2 **Cell proliferation assay.** A. Examples for Ki-67 staining in control SiRNA, Cdc42 and TCL transfected cells. The ratio of Ki-67 positive cells in control, Cdc42 and TCL siRNA transfected cells were not significantly different.
(TIF)
