# Supplementary material for: Rho GTPases and Regulation of Cell Migration and Polarization in Human Corneal Epithelial Cells
Source: PLoS One. 2013 Oct 10;8(10):e77107. doi: 10.1371/journal.pone.0077107 (PMC3795020; doi:10.1371/journal.pone.0077107)
Supplement: File S1 — File includes Supplementary Material and Method and Tables S1, S2, and S3. (DOCX) [file pone.0077107.s001.docx]

Supporting Information

**Supplementary Material and Method**:

**Cell cycle Assay**: Dominant-negative plasmids or siRNA were transfected into HCET cells as described in “Transfection by Electroporation”. Cells were seeded into 6-well plate and cultured in DMEM/F12 with 5% FBS for 24hrs. After that, cells were trypsinized and fixed in 70% enthanol. Cells were stained with Guava Cell Cycle reagent (Millipore, Billerica, MA, USA) according to the manufacturer’s instructions. Five thousands cells from each sample were analyzed. Cell cycle data were obtained using the Guava EasyCyte Plus flow cytometry system (Millipore, Billerica, MA, USA).

**Cell Proliferation Assay:** HCET cells transfected with control siRNA, Cdc42 targeting siRNA or TCL targeting siRNA were seeded into 8-well chamber slides (LabTek) for 24hrs, a scratch was made on the cell monolayer with a 1000ul pipette tip and cells were cultured for another 24hrs. After that, cells were subject to Ki-67 (Abcam) staining as described in the “**p21-activated kinase 4 (PAK4)** **immunofluorescence Staining”**

**Supplementary Figure Legend:**

**Figure S1**: Cell cycle Assay. **A**. HCET cells transfected with control plasmid, Cdc42 dominant-negative plasmid or TCL dominant-negative plasmid were stained with Guava Cell Cycle reagent and analyzed by Guava flow cytometry system. Percentages of cells at different cell cycle phase were shown. **B**. HCET cells transfected with control siRNA, Cdc42 targeting siRNA or TCL targeting siRNA were subjected to cell cycle assay as in **A**. In both conditions, cell cycles were not affected by Cdc42 or TCL inhibition.

**Figure S2:** Cell proliferation assay. A. Examples for Ki-67 staining in control SiRNA, Cdc42 and TCL transfected cells. The ratio of Ki-67 positive cells in control, Cdc42 and TCL siRNA transfected cells were not significantly different.

**Table S1** Primers used in RT-PCR

| **Gene** | Primers sequence |
| --- | --- |
| Cdc42 | F: 5’GTTCCCCATCTGGTGCTCTTAG3’  R: 5’CACCACCCCTCGTATTTCCTCT3’ |
| Chp (Wrch2) | F: 5’ GCCCGAGATCCGCACGCACAAC3’  R: 5’ GGCCCATGTCCCCCGAGTGTTC3’ |
| Rac1 | F: 5’ ATGCAGGCCATCAAGTGTGT3’  R: 5’TTACAACAGCAGGCATTTTC3’ |
| Rac2 | F: 5’CATCCGGGCCGTGCTGTGC3’  R: 5’AGGCGTGGGGTGGGACTGGA3’ |
| Rac3 | F: 5’TGAGCGGCTGCGGGACAAG3’  R: 5’CTCGGGGCACAAGGAACAGAACTC3’ |
| RhoA | F: 5’GGCTGCCATCCGGAAGAAACTG3’  R: 5’ATGTACCCAAAAGCGCCAATCCTG3’ |
| RhoD | F: 5’CCAGGCCGCGGGTGAGGAG3’  R: 5’TTTGGTGGCGCCCGTCTGTTGC3’ |
| Rif | F: 5’ ACGACACGGCCGGGCAAGAAGA3’  R: 5’GGGGCAGCCTGGAGGGGAGTTT3’ |
| TC10 | F: 5’GATGCTCAAGTGCGTGGTGGTC3’  R: 5’TGCTAAAGTTTTGGGGTCATCTCG3’ |
| TCL | F: 5’GCAGGGGCAACGACGAGAA3’  R: 5’CTTGGGGTGGAAAATGGTGAGG3’ |

**Table S2** List of siRNAs used in HCET cell transfection

| **siRNA** | **Catalogue Number** | **Target sequence (5'-3')** |
| --- | --- | --- |
| Cdc42 | custom | AAAGACUCCUUUCUUGCUUGU.dTdT |
| TCL | M-010367-01-0005 | 1) CCUCUGAAUGCCCGAUUAU 2) CCCGUUUGCUGUAUAUGAA 3) UCGUAAACCCUGCCUCUUA 4) AGAAACCUCUCACUUACGA |

**Table S3**: Post Hoc Tests for significance of differences of each condition in comparison to mock-transfected (control) HCET cells.

| **Paired comparisons against control group (Post Hoc Tests)** | **P value (Post Hoc tests)** |
| --- | --- |
| Cdc42 | 0.049* |
| TCL | 0.851 |

* Bonferroni adjusted p value < 0.05
