# Supplementary figures and images for: Rho GTPases and Regulation of Cell Migration and Polarization in Human Corneal Epithelial Cells
Source: PLoS One. 2013 Oct 10;8(10):e77107. doi: 10.1371/journal.pone.0077107 (PMC3795020; doi:10.1371/journal.pone.0077107)

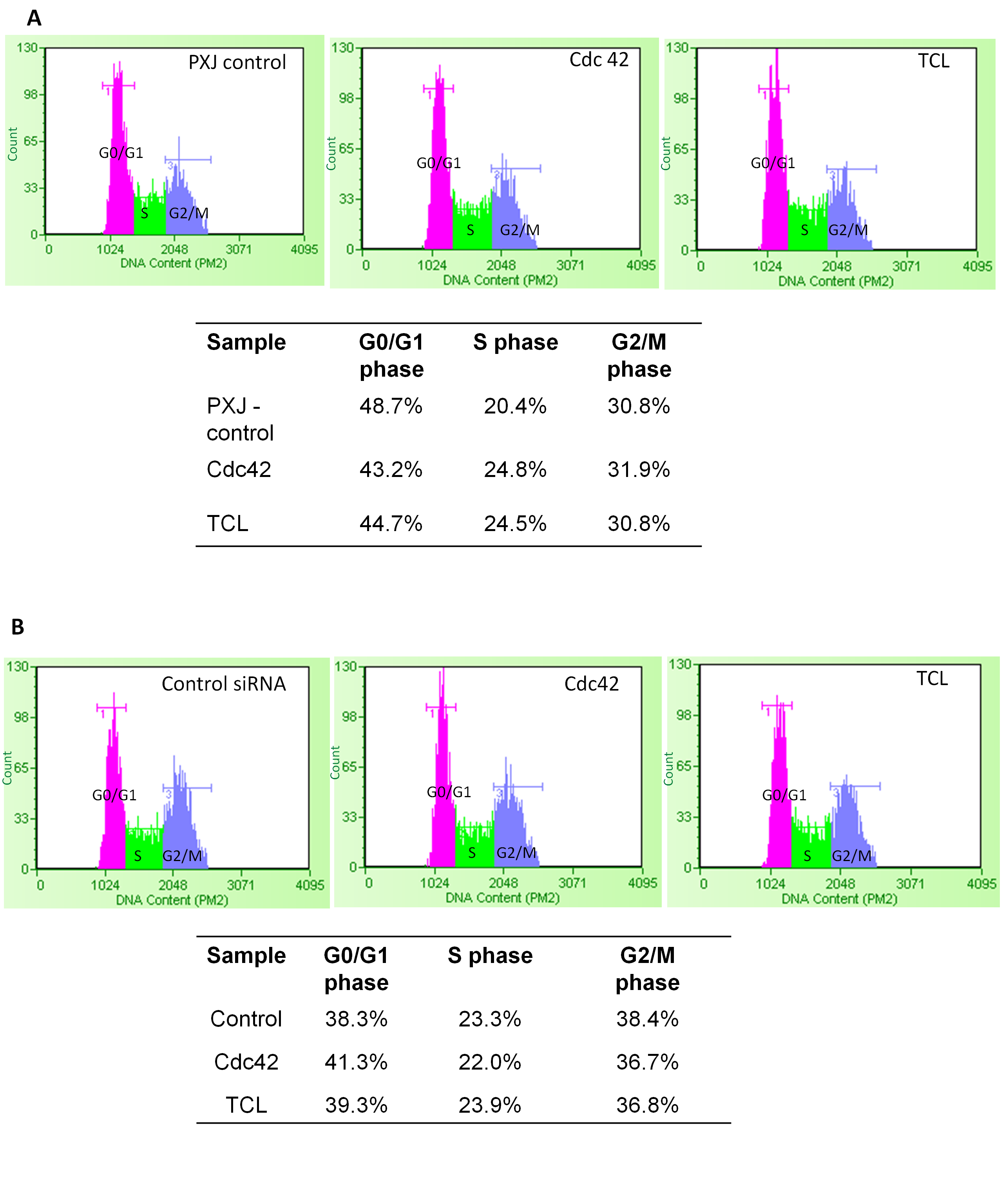

Supplement: Figure S1 — Cell cycle Assay. A. HCET cells transfected with control plasmid, Cdc42 dominant-negative plasmid or TCL dominant-negative plasmid were stained with Guava Cell Cycle reagent and analyzed by Guava flow cytometry system. Percentages of cells at different cell cycle phase were shown. B. HCET cells transfected with control siRNA, Cdc42 targeting siRNA or TCL targeting siRNA were subjected to cell cycle assay as in A. In both conditions, cell cycles were not affected by Cdc42 or TCL inhibition. (TIF) [file pone.0077107.s002.tif]

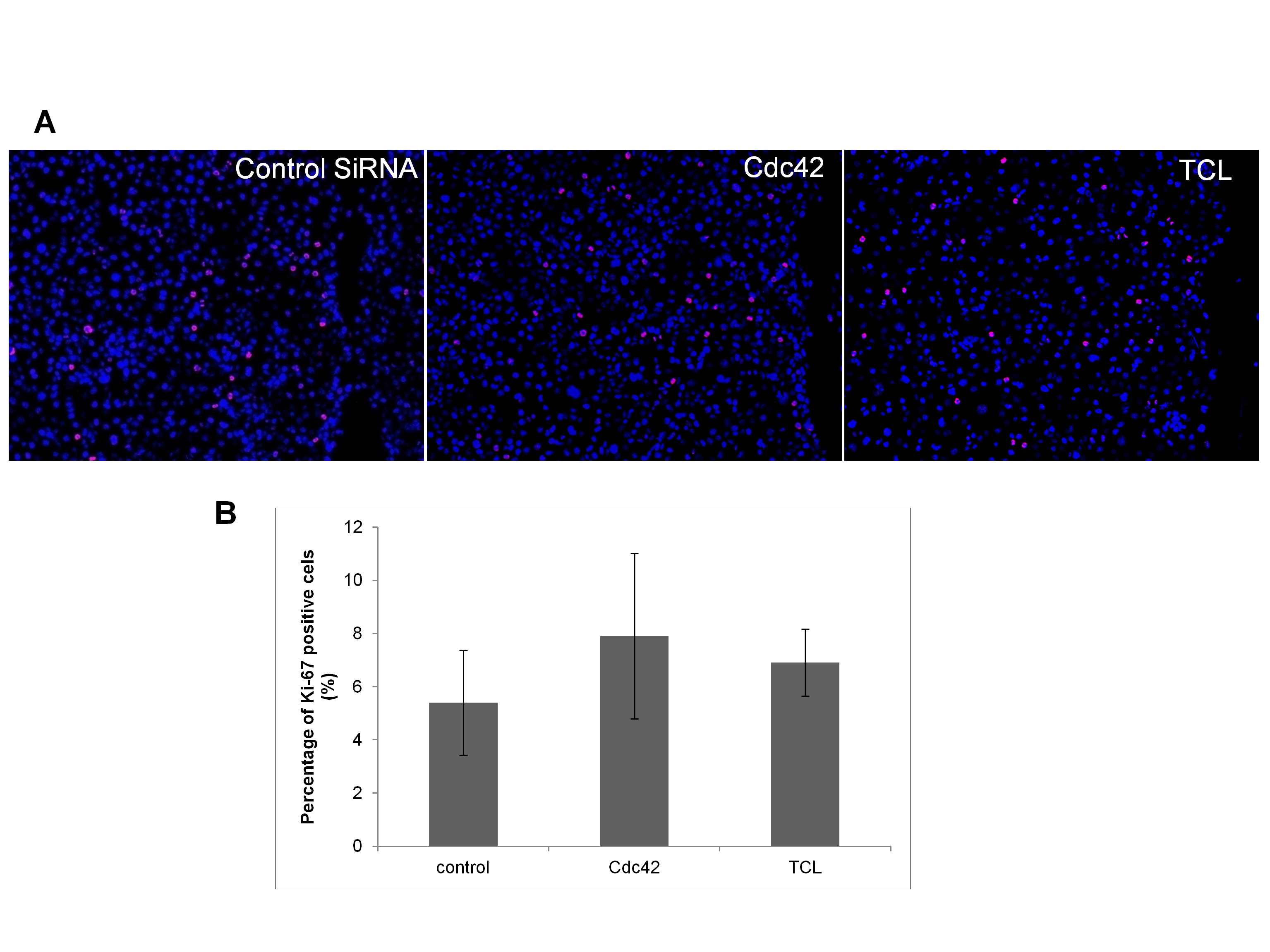

Supplement: Figure S2 — Cell proliferation assay. A. Examples for Ki-67 staining in control SiRNA, Cdc42 and TCL transfected cells. The ratio of Ki-67 positive cells in control, Cdc42 and TCL siRNA transfected cells were not significantly different. (TIF) [file pone.0077107.s003.tif]
